# 3D semiconducting nanostructures via inverse lipid cubic phases

**DOI:** 10.1038/s41598-017-06895-5

**Published:** 2017-07-25

**Authors:** M. R. Burton, C. Lei, P. A. Staniec, N. J. Terrill, A. M. Squires, N. M. White, Iris S. Nandhakumar

**Affiliations:** 1 School of Chemistry, University of Southampton, Southampton, SO17 1BJ UK; 2 Electronics and Computer Science, University of Southampton, SO17 1BJ Southampton, UK; 3 Department of Chemistry, University of Reading, Reading, RG6 6AD UK; 4 Diamond Light Source, Diamond House, Harwell Science and Innovation Campus, Didcot, Oxon OX11 0DE UK

## Abstract

Well-ordered and highly interconnected 3D semiconducting nanostructures of bismuth sulphide were prepared from inverse cubic lipid mesophases. This route offers significant advantages in terms of mild conditions, ease of use and electrode architecture over other routes to nanomaterials synthesis for device applications. The resulting 3D bicontinous nanowire network films exhibited a single diamond topology of symmetry *Fd3m* (Q_227_) which was verified by Small angle X-ray scattering (SAXS) and Transmission electron microscopy (TEM) and holds great promise for potential applications in optoelectronics, photovoltaics and thermoelectrics.

## Introduction

Over the past few years the emergence of synthetic strategies and fabrication technologies that afford control over the architecture of matter at nanometer length scales has provided a new tool kit for engineering functionality in materials. In particular the three-dimensional (3D) nanostructuring of metals and semiconductors has resulted in enhanced optical, magnetic and electronic properties yielding new devices and device concepts. 3D nanowire networks represent a good example and are of great technological importance for many applications including energy harvesting^1^, sensors^2^, energy storage^3, 4^, solar cells^5^, optoelectronics^6^ and electrocatalysis^7^. Hence there is an urgent need for developing approaches to their fabrication. Most of the methods reported to-date involve the use of hard templates such as porous alumina membranes (AAO)^8^ or polymeric substrates such as e.g. polycarbonate^9^ that act as templates for (either chemical or electrochemical) deposition of the respective material. The main limitations associated with these approaches is that these templates require multiple and often complex fabrication steps and can only be removed in either acids, sodium hydroxide (NaOH) or organic solvents such as dichloromethane or dimethylformanide^7, 10^ which raises safety and environmental concerns. These limitations can be overcome by using soft-templating approaches such as diblock copolymers^11^ or amphiphilic surfactants^12^. Diblock co-polymers for example have been used to produce gyroid networks of titania for applications in hybrid heterojunction solar cells^13^ and dye-sensitised solar cells^5^. The fabrication of diblock co-polymer templates however also involves synthetically complex steps and above all is very time-consuming, often taking several days^5, 13^. This has prompted us to explore whether inverse cubic lipid mesophases could provide an alternative fabrication method for 3D semiconductor nanostructures.

In the present study highly ordered 3D bicontinuous nanowire network films of the semiconductor compound bismuth sulphide (Bi_2_S_3_) were prepared by electrodeposition through inverse lipid cubic phases of phytantriol at room temperature under mild aqueous conditions. A combination of SAXS and TEM in conjunction with Matlab simulations and Fast Fourier Transforms (FFTs) has been utilised to verify the formation of a single diamond topology bismuth sulphide films with symmetry *Fd3m* (Q_227_) from a double diamond phytantriol template of symmetry Q_224_.

Bi_2_S_3_ has attracted considerable attention due to its potential for applications within photovoltaics^14, 15^ and thermoelectric coolers^16^ given its direct band gap of 1.3 eV and positive photoconductivity. Thin films of Bi_2_S_3_ have been prepared by either chemical bath deposition^16^ or spray pyrolysis^17^, whilst nanowires as well as nanorod films were grown by hydrothermal synthesis^18^ or solventless thermolysis^19^. These methods necessitate elevated temperatures for successful synthesis and do not produce ordered and highly interconnected 3D nanostructures that are suitable for incorporation into devices. Whilst the electrochemical fabrication of Bi_2_S_3_ has been reported, literature coverage is extremely limited^20–23^ and to the best of our knowledge there are currently no studies on the formation of ordered nanowire networks of Bi_2_S_3_ films from bicontinuous cubic lipid phases. The use of inverse cubic lipid mesophases for the preparation of nanostructured semiconductors such as Bi_2_S_3_ has a number of distinct advantages over other routes to nanomaterial synthesis as illustrated in this study: Seminconductor compounds as can be deposited at room temperature, under chemically mild aqueous conditions. The templating process faithfully replicates the size and geometry of the template whilst the templates themselves can be prepared, and removed post-templating, by simple one-step processes using water or ethanol, making the entire process green, cost-effective and compatible with a range of substrate materials for device fabrication. The ability to fabricate 3D semiconductor nanostructures such as Bi_2_S_3_ with a bicontinuous cubic morphology will be highly attractive for many applications such as photovoltaics, thermoelectrics and optoelectronic as these materials have displayed enhanced charge transfer^24^ and tunable phononic^25^ and photonic bandgaps^26^.

**Figure 1:**
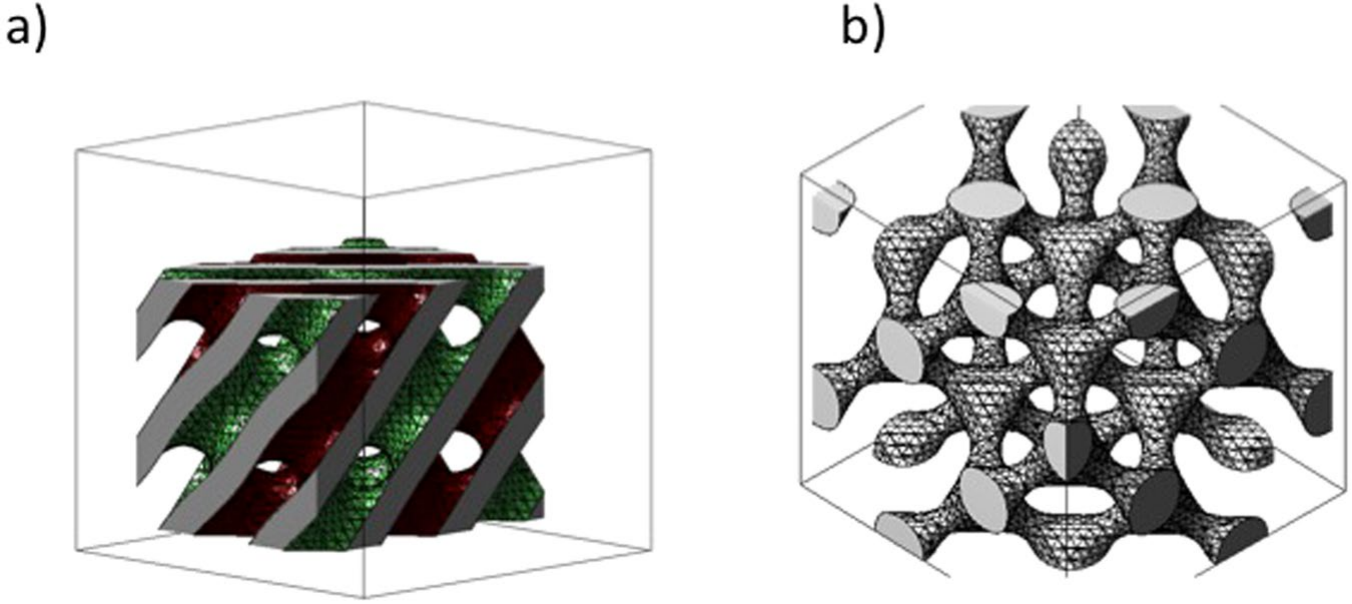
Schematic representation of (**a**) bicontinuous double diamond Q_II_ cubic phase of phytantriol displaying the two water channels in green and red and (**b**) single diamond bismuth sulphide nanostructure generated by MATLAB.

The phase diagram of phytantriol has previously been studied in detail by Baraukas *et al*.^27^ and there are three symmetries of bicontinuous cubic structures that could be identified: the gyroid (G), double diamond (D), and primitive (P) surfaces corresponding to the space groups Ia3d (Q_230_), Pn3m (Q_224_), and Im3m (Q_229_), respectively. For illustration, the bicontinuous double diamond phase is depicted in Fig. 1: This contains a single lipid bilayer on either side of which lie branching networks of nanometre-sized water channels.

## Methods

Nanowire network films of bismuth sulphide were prepared by potentiostatic electrodeposition at a potential of −0.4 V vs SCE from an electrolyte containing 100 mM Bi(NO_3_)_3_, 100 mM Na_2_S_2_O_3_ and 200 mM EDTA through phytantriol modified gold (Au) DVDs (Delkin Devices). Bismuth sulphide electrolytes were prepared by mixing the bismuth and sulphide precursors along with EDTA in dissolved water (Merck Millipore Milli-Q Gradient A10 Ultrapure water purification system, 18.2 MΩ cm) over night. The solution was then left to settle for 2 days to allow a formed black precipitate to settle. A clear electrolyte solution was then separated and bubbled through with Argon for a minimum of 20 minutes, to remove dissolved oxygen. The two plastic layers of the DVD were separated using mechanical force. The exposed gold layer disc was cut into 1.5 cm × 1.5 cm using scissors. Cu tape was used to make an electrical contact and polyimide tape was used to isolate a 10 mm × 10 mm working electrode area. The working electrode was dip coated in a solution of phytantriol and ethanol 1:2 by weight, allowed to dry for 30 minutes prior to being immersed in the electrolyte and was allowed to hydrate for 30 minutes in the electrolyte prior to electrodeposition. Films were deposited potentiostatically at a potential of −0.4 V vs. SCE for 4 h. A saturated calomel reference electrode and a Pt gauze counter electrode were used. Post deposition films were washed for a minimum of 30 minutes in ethanol.

A Zeiss EVO LS25 ESEM scanning electron microscope (SEM) with an Oxford Instruments energy dispersive x-ray spectrometer attachment was used to determine surface topography and composition of the Bi_2_S_3_ deposits. Accelerating voltages up to 15 KV were applied and images recorded with a secondary electron detector. Samples were mounted as deposited (on Au DVDs) on an 18 mm SEM clip specimen mount (agar scientific). XRD Grazing incidence scans, where the source is held at a constant shallow angle to the sample and the detector is scanned through theta values, were conducted on the nanotemplated films. A Rigaku SmartLab X-Ray diffractometer was used to obtain diffraction patterns. The Rigaku Smartlab uses Cu Kα radiation with a wavelength of 1.5406 Å. Small angle x-ray scattering measurements were carried out at Diamond light source on beamline I22 with beam energy and size of 12.4 KeV and 320 µm × 80 µm respectively. A Pilatus 2 M detector^28^ was used to collect data over the q range of 0.05 Å^−1^–0.30 Å^−1^. Calibration was achieved by using a silver behenate^29^ sample. Phases were indexed by assigning Bragg peaks to known phases. Samples were prepared for transmission electron microscopy by scraping small sections of nanotemplated films with a scalpel over 400 mesh copper transmission electron microscopy grids (Sigma Aldrich). A FEI Tecnai T12 transmission electron microscopy was used to acquire transmission electron microscopy images.

## Results and Discussion

**Figure 2:**
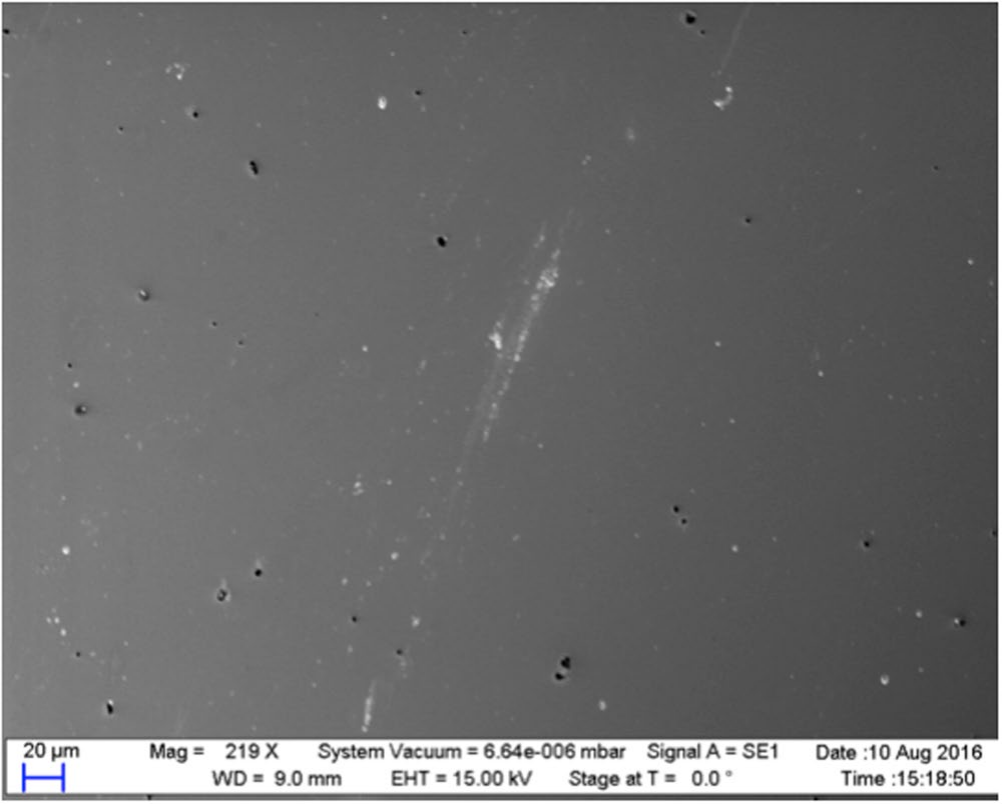
Scanning electron microscopy images of a bismuth sulphide film electrodeposited through a phytantriol template from an electrolyte solution containing 100 mM Bi(NO_3_)_3_, 100 mM Na_2_S_2_O_3_ and 200 mM EDTA in deionised water. The film was deposited potentiostatically at a potential of −0.4 V vs. SCE for 4 hours.

The surface morphology of the phytantriol templated bismuth sulphide films was investigated by SEM and a representative image is shown in Fig. 2 which indicates a mostly smooth surface morphology with some pin holes being present. EDX of the phytantriol templated bismuth sulphide films showed a reproducible composition of 40.1 (±5.5)% Bi and 59.9 (±5.5)% S, resulting in a stoichiometry of Bi_2_S_3_.

**Figure 3:**
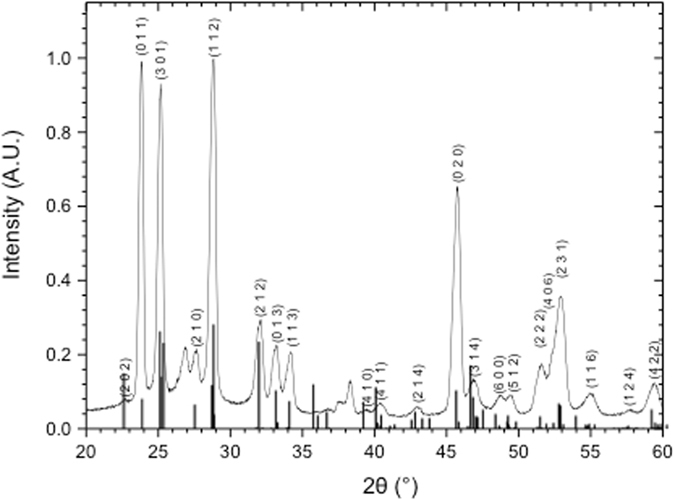
Grazing incidence x-ray diffraction scan of a bismuth sulphide film electrodeposited through a phytantriol template with superimposed pdf card 9007375 for Bismuthinite from the crystallographic open database.

Figure 3 shows a representative grazing incidence X-ray diffraction scan of an electrodeposited Bi_2_S_3_ film through phytantriol. The diffraction pattern has been indexed in accordance with the pdf card 9007375 for Bismuthinite from the crystallographic open database (COD) which has been superimposed in Fig. 3. There is good agreement between the experimental and theoretical data which indicates that indeed bismuthinite or bismuth sulphide of stoichiometry Bi_2_S_3_ has formed.

**Figure 4:**
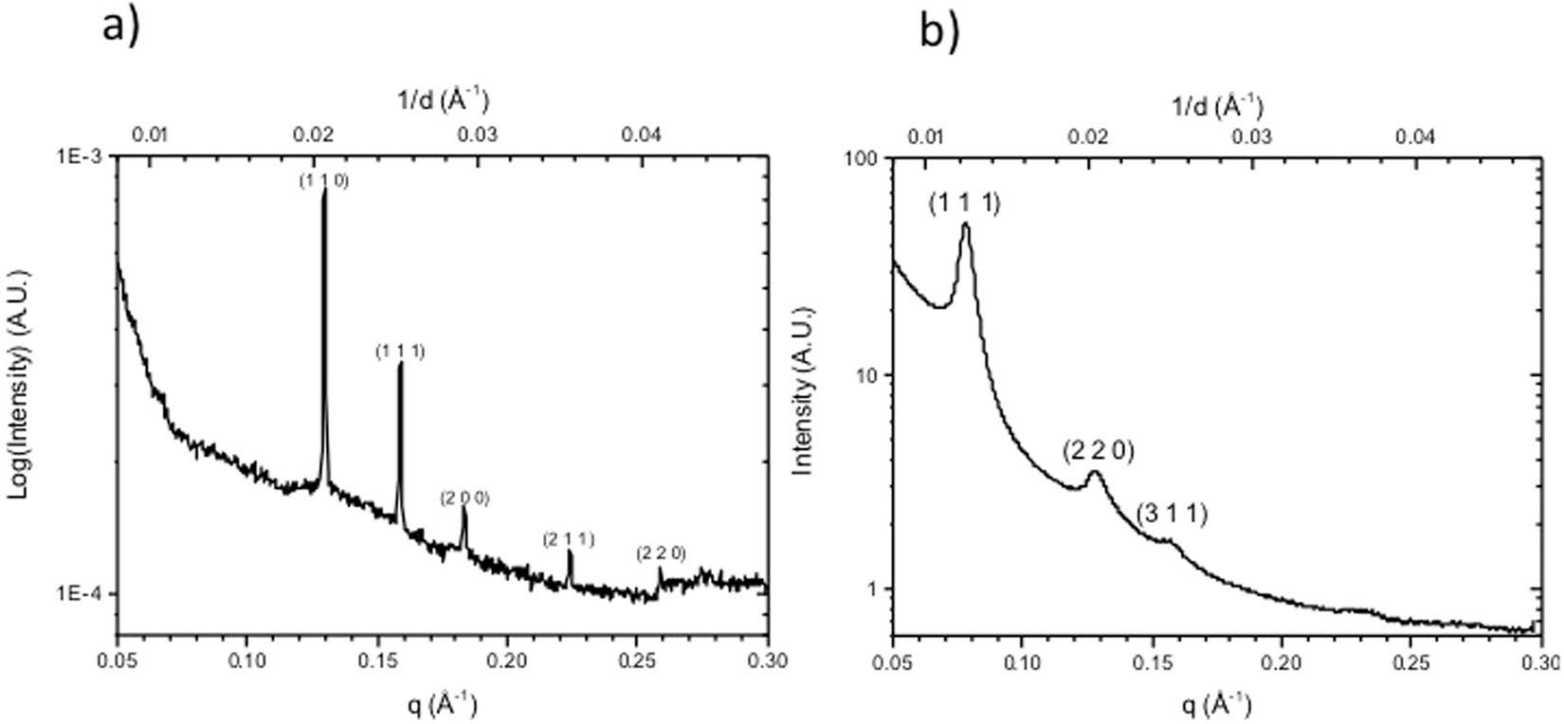
(**a**) 1D integrated SAXS patterns of a phytantriol coated gold foil immersed in 100 mM Bi(NO_3_)_3_, 100 mM Na_2_S_2_O_3_ and 200 mM EDTA in deionised water and (**b**) of a bismuth sulphide film electrodeposited through a phytantriol template. The film was deposited potentiostatically at a potential of −0.4 V vs. SCE for 4 hours.

Small angle X-ray scattering (SAXS) in transmission mode and Transmission Electron Microscopy (TEM) were employed to verify and visualize the nanostructures. Figure 4a and b show 1D SAXS radial profiles of the phytantriol modified gold electrode surface prior to electrodeposition (4a), immersed in an electrolyte solution of 100 mM Bi(NO_3_)_3_, 100 mM Na_2_S_2_O_3_ and 200 mM EDTA, and following electrodeposition after removal of phytantriol (4b).

Prior to electrodeposition we can distinguish Bragg peaks with relative positions for 1/d in the ratio √2: √3: √4: √6: √8 which are representative for the Q224 bicontinuous cubic lattice of phytantriol, with a lattice parameter of 68.9 ± 0.9 Å. Following electrodeposition and subsequent removal of phytantriol the 1D SAXS radial profiles reveal a sequence of three distinct and well-resolved Bragg diffraction peaks with 1/d spacings in the ratio of √3: √8: √11 which are characteristic of a single diamond topology of symmetry *Fd3m* (Q_227_), with a lattice parameter of 139.6 ± 3.2 Å. This corresponds to approximately double the lattice parameter of phytantriol in the electrolyte (68.9 ± 0.9 Å) and is consistent with electrodeposition occurring in one water channel of the double diamond structure, resulting in a single diamond nanostructure^30^ with a doubled lattice parameter due to a reduction in symmetry as shown in Fig. 1(b).

**Figure 5:**
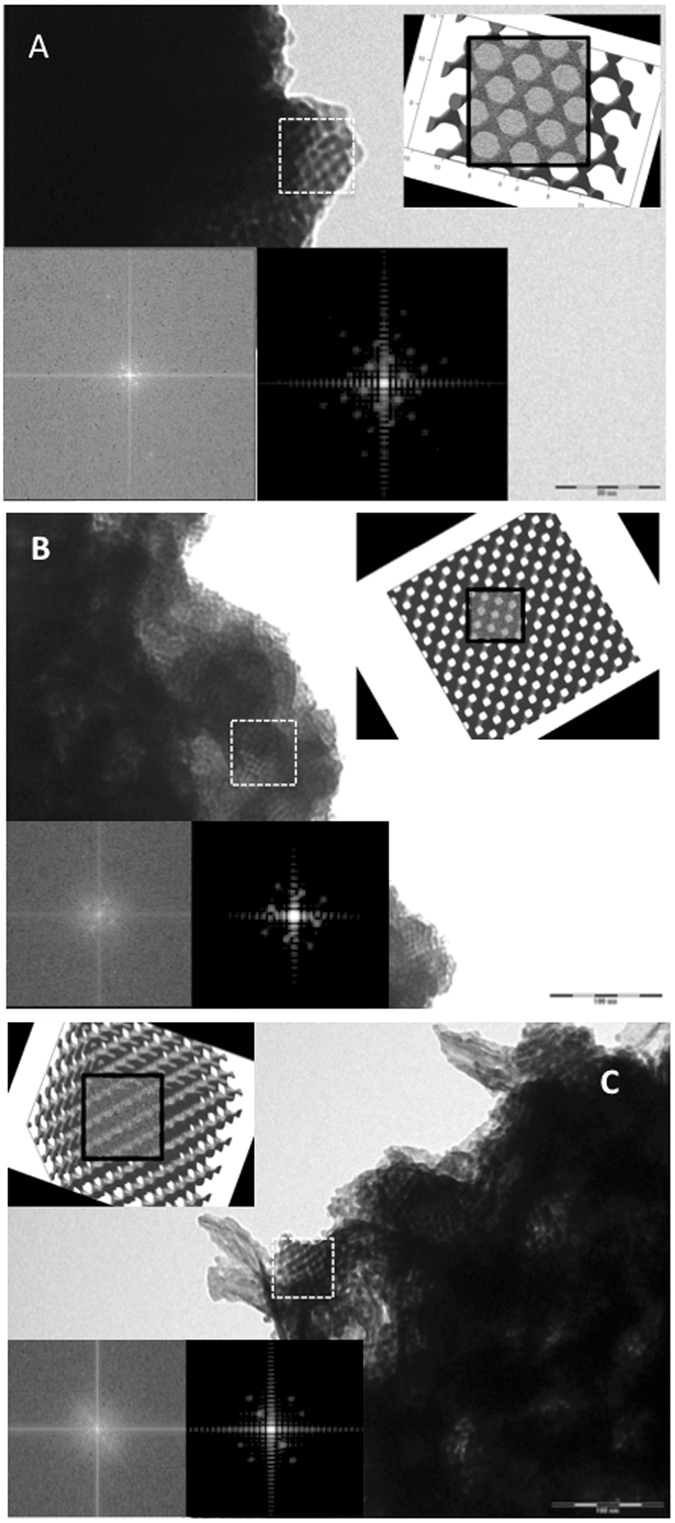
TEM images with corresponding Matlab projections for a single diamond topology nanostructure viewed along the following directions: (**A**) [110], (**B**) [100] and (**C**) non-integer direction perpendicular to [111]. FFTs for each TEM image (bottom left) have been included. For comparison, the corresponding MATLAB projection is shown (top right), with the highlighted rectangular section (with Gaussian noise added artificially) used to produce the corresponding simulated FFT (bottom right).

To provide further evidence TEM images in combination with MATlab projections are shown in Fig. 5 which reveal a well ordered nanostructure. The MATLAB projections with the highlighted rectangular section (with Gaussian noise added artificially) are then used to produce the corresponding simulated FFT (bottom right) which can be directly compared against the FFTs of the TEM image (bottom left). These are in excellent agreement and also reveal the cubic symmetry of the single diamond structure.

From the MATlab projections as well as the TEM images the following lattice parameters can be estimated: a) 125 Å, b) 109 Å and c) 124 Å. The lattice parameters based on the simulations therefore matched with a lattice parameter of a similar order of magnitude as those determined by SAXS (139.6 ± 3.2 Å), however, these were smaller by 25%. This may be due to actual shrinkage of the bismuth sulphide films after detachment from the substrates. Nevertheless it can be concluded that within experimental error as evidenced by SAXS and TEM data the formation of a single diamond morphology in nanostructured bismuth sulphide has occurred.

## Conclusions

We have demonstrated for the first time that inverse lipid cubic mesophases can act as suitable templates for the room temperature production of highly ordered 3D semiconductor compound nanostructures (bismuth sulphide) as evidenced by SAXS and TEM. Fabrication via this route can be carried out in one step without the need for elaborate and complex synthetic steps resulting in well-ordered materials that are attached to an electrode surface. Hence this therefore represents an important step forward towards the fabrication of nanostructured 3D semiconductors for device applications.
